# Quantitative perfusion cardiac magnetic resonance imaging: same same but different

**DOI:** 10.1007/s10554-025-03486-8

**Published:** 2025-08-18

**Authors:** Roel Hoek, Ruurt A. Jukema, Henk Everaars, Pepijn A. van Diemen, Roel S. Driessen, Marco J. W. Götte, Paul Knaapen

**Affiliations:** Department of Cardiology, Amsterdam University Medical Center, Vrije Universiteit Amsterdam, Amsterdam, the Netherlands

**Keywords:** Coronary artery disease, Positron emission tomography, Quantitative perfusion cardiac magnetic resonance imaging

In patients with suspected chronic coronary syndromes (CCS) and a moderate to high pre-test likelihood of obstructive coronary artery disease (CAD), the 2024 ESC Guidelines for the management of CCS recommend the use of quantitative myocardial perfusion imaging for diagnostic purposes [1]. However, these guidelines have not yet endorsed the use of quantitative perfusion cardiac magnetic resonance (QP-CMR) imaging for clinical purposes, given the lack of standardized protocols and software. The influence of different CMR acquisition protocols has been studied extensively [2]. However, the impact of specific post-processing software tools on myocardial blood flow (MBF) quantification remains largely unexplored, particularly in comparison with the gold standard of MBF quantification, [^15^O]H_2_O positron emission tomography (PET). In this regard, understanding the effect of software variations on MBF measures and subsequent diagnostic performance assessment could help unravel key obstacles to the clinical implementation of QP-CMR. Therefore, we aimed to investigate the correlation between MBF values derived from two different QP-CMR software tools, their relationship with [^15^O]H_2_O PET-derived MBF, and whether discrepancies between the tools influence diagnostic performance for detecting ischemia.

We conducted a post-hoc study of the PACIFIC-2 trial, in which 189 symptomatic CCS patients with prior myocardial infarction and/or percutaneous coronary intervention underwent both dual-sequence, single-bolus stress perfusion CMR and quantitative [^15^O]H_2_O PET. The current study included all patients that had been included in two prior studies using two different QP-CMR software tools [3, 4]. The first applied QP-CMR software tool (QP_1_) was Circle Cardiovascular Imaging 42 software (cvi42; Circle Cardiovascular Imaging Inc., Calgary, Canada); the second (QP_2_) was MASS (version 2017- Exp, Leiden, the Netherlands). The analysis methodologies of all patients using both software tools, as well as the CMR and PET scanning protocols, have been published previously [3, 4]. Mean QP_1,2_ and PET MBF values (mL min^−1 ^g^−1^) are reported at a vascular territory level, and were compared using paired samples t-test. Correlation between stress MBF values was performed using Pearsons correlation. Areas under the receiver operating characteristics curve (AUCs) were generated for mean vascular stress MBF of QP_1,2_ to detect PET-defined ischemia (PET: stress MBF ≤ 2.3 mL min^−1 ^g^−1^ in ≥ 2 adjacent segments within a vascular territory), and compared using the DeLong method. Our study complied with the Declaration of Helsinki and was approved by the local medical ethical committee. All patients provided written informed consent.

We included 50 patients (mean age 63.5 ± 8.4 years, 26% female) with 149 vascular territories. The mean vascular territory rest and stress MBF were lower for QP_1_ as compared to QP_2_ (rest: 0.98 ± 0.18 vs. 1.15 ± 0.29, *P* < 0.001; stress: 1.68 ± 0.50 vs. 3.04 ± 0.87, *P* < 0.001). PET rest MBF (0.96 ± 0.29) was equal to QP_1_ (*P* = 0.332) and lower than QP_2_ (*P* < 0.001). Conversely, PET stress MBF (3.09 ± 0.91) was higher than QP_1_ (*P* < 0.001) and equal to QP_2_ (*P* = 0.516). The correlation between QP_1,2_ and PET stress MBF was poor for both QP_1_ (*r* = 0.32, *P* < 0.001) and QP_2_ (*r* = 0.27, *P* < 0.001) (Fig. 1A, upper panel). When using PET as a reference method to indicate ischemia on a vascular territory level, AUCs were similar for QP_1_ (0.70 [95% confidence interval (CI): 0.62–0.77]) and QP_2_ (0.69 [95% CI: 0.61–0.76], *P* = 0.817) (Fig. 1B). Interestingly, the correlation between QP_1_ and QP_2_ derived stress MBF was also found to be poor (*r* = 0.27, *P* < 0.001), despite both showing similar correlation and diagnostic performance when compared to PET (Fig. 1A, lower panel). The poor correlation between QP-CMR and PET derived stress MBF is similar to our previously published correlation coefficients [3, 4]. Both QP_1_ and QP_2_ had a modest but similar diagnostic performance for detecting [^15^O]H_2_O PET derived ischemia, which is regarded as the gold standard for quantification of MBF. This agreement in correlation and diagnostic performance could indicate similarity between the MBF patterns of the different QP-CMR modalities. However, a correlation of 0.27 between QP_1_ and QP_2_ suggests the opposite, postulating that while these values may appear the same, they are in fact quite different.

Our results are in line with a prior QP-CMR study in which the authors found a similar weak correlation between two QP-CMR tools (*r* = 0.29) [5]. However, yielding almost identical AUC values has not been previously demonstrated. As such, our results suggest that both software tools are identifying similar ischemic patterns despite their difference in MBF values, but also implies that quantification is not yet accurate. We have to acknowledge that our results might be impacted by the used CMR acquisition protocol or studied patient population. Nevertheless, the findings of this post-hoc study underscore the recently published European guidelines, emphasizing the need for protocol- and software-specific cutoff values to diagnose ischemia. Future research including software optimization is essential to accurately quantify MBF using QP-CMR, and to ensure that inter-software QP-CMR MBF values not only appear similar, but are indeed similar.

**Fig. 1:**
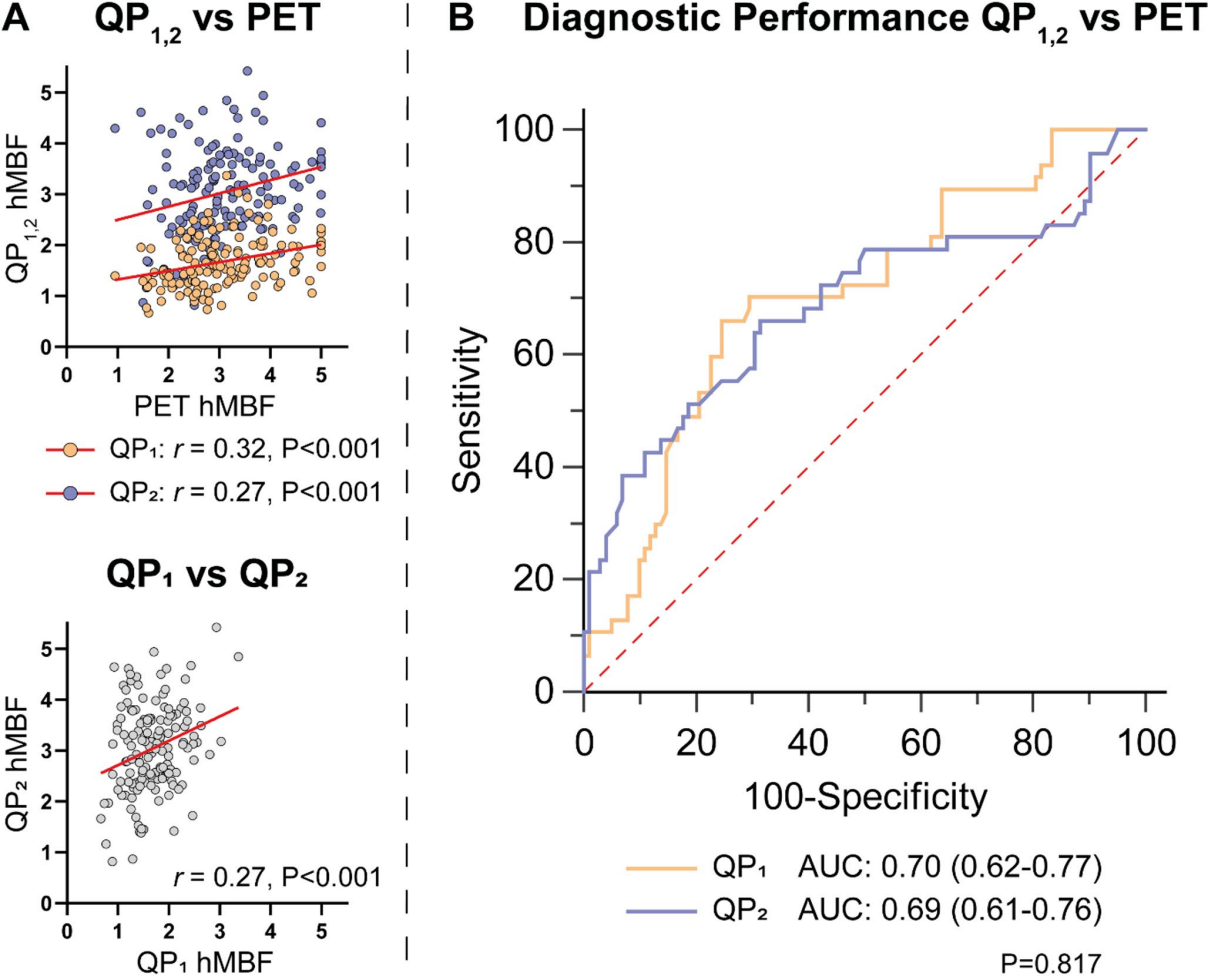
Correlation and diagnostic performance between QP_1,2_ and [^15^O]H_2_O PET AUC: Area under the receiver operating characteristics curve, hMBF: hyperemic myocardial blood flow, PET: positron emission tomography, QP: QP-CMR software tool

## Data Availability

No datasets were generated or analysed during the current study.

## Abbreviations

CAD: Coronary artery disease
CCS: Chronic coronary syndromes
MBF: Myocardial blood flow
PET: Positron emission tomography
QP-CMR: Quantitative perfusion cardiac magnetic resonance imaging
